# Primary Ovarian Malignant Melanoma: A Case Report

**DOI:** 10.7759/cureus.84249

**Published:** 2025-05-16

**Authors:** Maria Zoi Bourou, Theodoros Panoskaltsis, Despoina Myoteri, Porfyrios Korompelis, Panagiotis Vakas

**Affiliations:** 1 Second Department of Obstetrics and Gynecology, National and Kapodistrian University of Athens Medical School, Aretaieio University Hospital, Athens, GRC; 2 Department of Pathology, National and Kapodistrian University of Athens Medical School, Aretaieio University Hospital, Athens, GRC; 3 Department of Gynecologic Oncology, Queen Elizabeth Hospital, Gateshead, GBR

**Keywords:** adnexal mass, case report, pelvic organ prolapse, primary ovarian malignant melanoma, rare tumors

## Abstract

Primary ovarian melanoma (POM) is an exceedingly rare and aggressive form of melanoma that arises in the ovaries, distinct from metastatic ovarian melanoma, which is far more common. The rarity of this condition, coupled with its clinical similarities to other types of ovarian malignancies, makes it a challenging entity to diagnose and manage. We present the case of a 56-year-old woman who underwent a laparoscopically assisted vaginal hysterectomy and bilateral salpingo-oophorectomy due to pelvic organ prolapse and a left adnexal mass. The final histology showed a POM. She received adjuvant immunotherapy, and a year after the initial diagnosis, the patient remains alive and well, without any signs of recurrence or distant disease.

## Introduction

The incidence of malignant melanomas has significantly increased over the last 30 years. Melanomas of the female reproductive system are infrequent tumors, accounting for 3% of malignant tumors in women [1]. They are usually presented in the vulva, vagina, and cervix and less commonly in the uterine body and ovary [2]. Primary ovarian melanoma (POM) is an extremely rare malignancy of the ovary [1]. The first case was described by Andrews in 1901 [3,4] and, to the best of our knowledge, only 49 cases of POMs were published in the literature [3]. Patients with POM often present with non-specific abdominal symptoms, including pain, bloating, and changes in bowel habits [4]. Diagnosis of POM is complicated by its infrequency and the similarity of its presentation to metastatic malignant melanoma [5]. Histologically, it displays the classic features of melanoma, including atypical melanocytes, varying degrees of pigmentation, and invasive growth patterns [6]. Because of the rarity of these tumors, there are no specific recommendations, so treatment is often decided individually based on the therapeutic regimen for ovarian cancer or other types of melanomas [4]. This article was previously presented as an e-poster at the ESGO (European Society of Gynaecological Oncology) 2025 Congress on February 20, 2025.

## Case presentation

**Figure 1:**
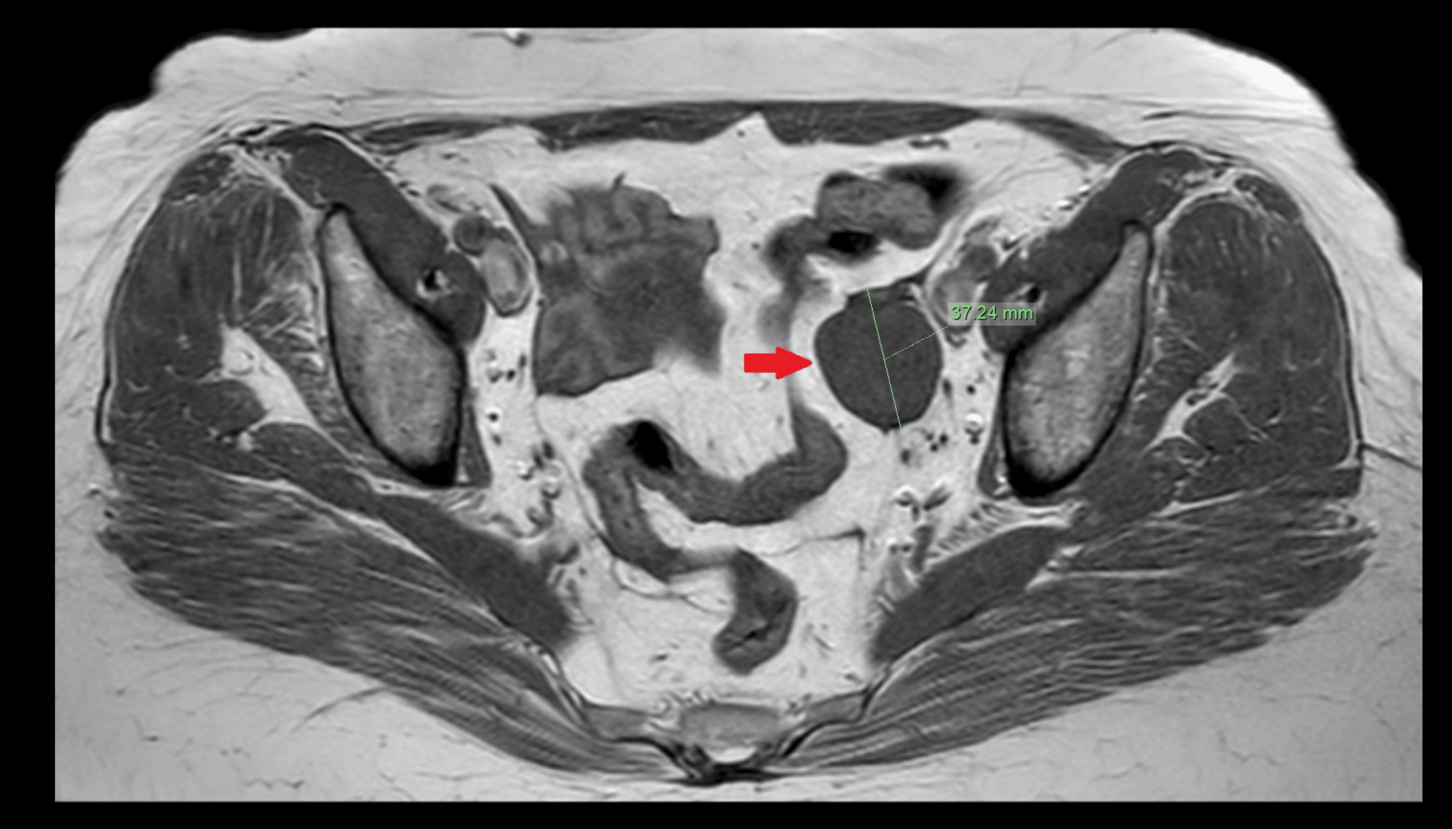
The red arrow highlights a 4-cm-diameter left adnexal mass with low signal on MRI. MRI, magnetic resonance imaging

The patient was a 56-year-old, gravida 3 para 3, post-menopausal woman, who presented with pelvic organ prolapse. During pre-operative investigation with transvaginal ultrasound scan, an incidental finding of a 4 cm left adnexal mass was identified. The mass was solid with distal vascularization, and the risk of ovarian malignancy algorithm (ROMA) score was normal. A pelvic MRI (Figure 1) confirmed the finding, and the pelvic mass was characterized as an ovarian fibrothecoma by the reporting radiologists. All the tumor markers, including CA125, CA 19-9, AFP, and βhCG, were within the normal range.

The patient was admitted to the Second Department of Obstetrics and Gynecology at Aretaieio University Hospital (Athens, Greece) in January 2024. A laparoscopically assisted vaginal hysterectomy (LAVH), bilateral salpingo-oophorectomy (BSO), and anteroposterior colporrhaphy were performed. During the operation, there was no evidence of peritoneal disease, and the left ovarian tumor was 5 cm in diameter, grayish with a bound appearance, and had a sub-hard constitution. Together with the uterus and the right adnexum, the left adnexum with the intact mass was removed through the vagina. The patient made an uneventful recovery, and she was discharged from the hospital two days after the operation.

**Figure 2:**
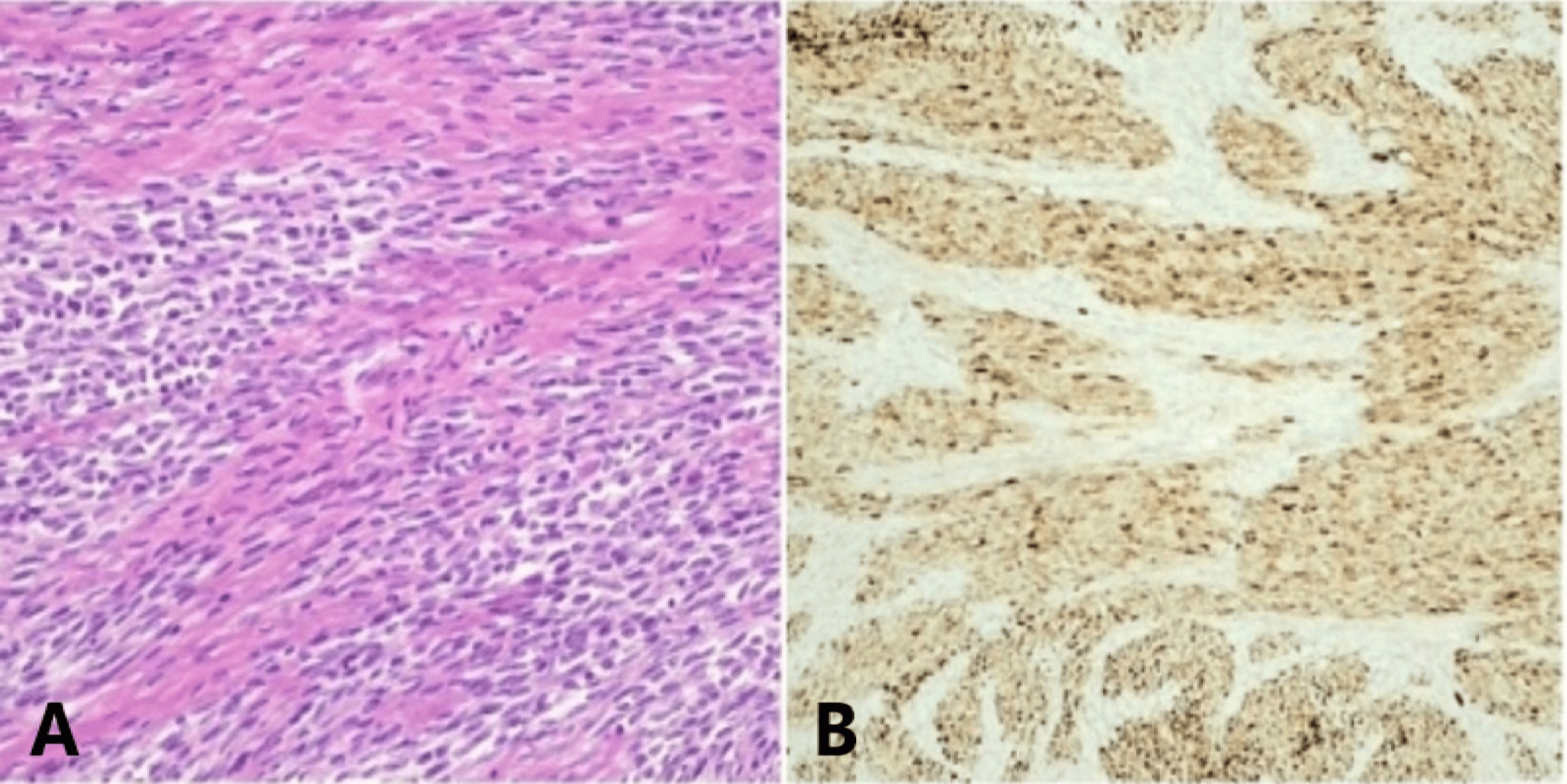
(A) Hematoxylin and eosin stain showing neoplastic spindle cells arranged in fascicles. (B) Immunohistochemically, tumor cells are diffusely positive for S-100 protein.

Final histology showed a highly cellular tumor of the left ovary. The cancerous cells were fusiform, exhibiting infrequent mitoses and elongated nuclei (Figure 2A). Extensive immunohistochemistry staining was performed, including Vimentin (+), S100 (+), Calretinin (+), SOX10 (−), HMB45 (−), Melan-A (−), Inhibin (−), ER (−), Pan-Keratin (−), CD99 (−), WT1 (−), SMA (−), Desmin (−), CD10 (−), PAX8 (−), CK7 (−), EMA (−), Synaptophysin (−), Chromogranin (−), NSE (−), CD3 (−), CD20 (−), GATA3 (−), p63 (−), CDX2 (−), Napsin A (−), TTF-1 (−), and no *BRAF* mutations were identified. The morphological and immunohistochemical findings were consistent of a fusiform ovarian melanoma (Figure 2B).

Following discussion at the gynecologic oncology tumor board meeting, further gynecological, dermatological, and opthalmological examinations were performed to exclude the possibility of a primary melanoma arising from another site. A brain MRI and a full body PET scan were also performed. All clinical and radiological examinations were negative and no other suspicious lesions were identified.

The tumor board recommended adjuvant systemic treatment. The patient received five cycles of immunotherapy with nivolumab, in combination with ipilimumab, under the care of a melanoma specialist medical oncologist. CT scans at six-monthly intervals showed no evidence of local recurrence or distant metastases. A year after, the patient remains alive and well.

## Discussion

POMs are much rarer compared to the metastatic ones [4]. Only a few number of cases have been reported in the literature since its first publication, and the majority of them developed against the backdrop of an ovarian cystic teratoma [3]. With an estimated incidence of 0.2%-0.8%, such a neoplastic transformation has been a very uncommon occurrence [7]. Normal ovarian tissue does not contain any melanocytic material. However, a cystic ovarian teratoma may contain melanocytes, particularly at the squamous epithelial basal layer [8].

Malignant ovarian tumors secondary to ovarian mature teratomas can present at any age. A 25-year-old primigravida patient with a pure ovarian melanoma and a child with a POM arising in the teratomatous component of a mixed-germ cell tumor are the youngest patients’ cases described in the literature [9,10]. More frequently, as in our case, POMs occur in post-menopausal women between the ages of 50 and 60 [4].

Due to the lack of unusual symptoms or radiological characteristics, the final histopathological diagnosis is made after surgery [11,12]. Occasionally, as the amount of melanin in the lesion is correlated with a hyperintense signal in T1-weighted images, MRI of the lesion may raise suspicion about its nature [5]. In our case, the radiological diagnosis of the ovarian tumor was that of a fibrothecoma, and the ROMA score was normal. Also, there is a long range of months to 18 years between the primary melanoma diagnosis and ovarian metastases [5]. Increased serum CA 19-9 levels have been suggested as a diagnostic tool for ovarian mature cystic teratoma, even though they are not pathognomonic [6]. This is particularly true if the melanoma develops on a background of a mature teratoma. However, tumor marker levels are typically not discriminatory. In our case, all tumour markers were negative.

The pathological diagnosis is extremely challenging because of the non-specific morphology of these lesions [13]. Histologically, diagnostic challenges also occur because tumors can be mistaken and have inconsistent appearances. Metastatic ovarian malignant melanoma is part of the different diagnosis [5,14], which should be distinguished from primary and secondary ovarian tumors such as lymphoma, undifferentiated carcinoma, primitive neuroectodermal tumor, and malignant sex cord stromal tumors [1]. Mixed ovarian neoplasms with parts of malignant melanoma have also been described in the literature [3,15]. A more definitive diagnosis may be established with the aid of immunohistochemistry, using melanocytic markers such as S-100, Melan-A, and HMB45 antibodies [4]. The most sensitive marker, S-100, is found in 95% of cases and is expressed in both the cytoplasm and the nucleus [5]. The most precise diagnostic criteria are produced by combining the more sensitive S-1OO with the more specific HMB45 [2]. 

Whole-exome sequencing in gynecologic mucosal melanomas (MMs), including the rare ovarian ones, often identifies mutations in *NRAS*, *NF1*, *KMT2D*, *MUC16*, and *KIT*. Nevertheless, they present a lower frequency of canonical *BRAF* mutations, making them less sensitive to BRAF/MEK inhibitors. MM lacks a UV mutational signature and has higher genomic instability. In contrast to cutaneous melanoma, it has lower levels of programmed death ligand 1 and tumor-infiltrating lymphocyte, which, in combination with the lack of a large tumor mutational burden, may lead to poorer response rates to immune checkpoint inhibitors [16].

Currently, there are no guidelines for the therapeutic management of POM because it is very uncommon and challenging to gather enough patients for clinical trials [4]. The mainstay of treatment for ovarian melanomas is still surgery [17,18]. Although a number of operations have been reported in the literature, the most frequent ones are BSO and hysterectomy [4]. Conservative treatment may be appropriate for young, pre-menopausal women who wish to maintain their fertility [18,19]. Resection of additional organs, such as omentectomy, pelvic and/or para-aortic lymphadenectomy, appendectomy, and primary debulking surgery, have also been reported [2,18]. Intravenous chemotherapy (usually platinum-based) and immunotherapy (interferon-alpha, vaccines, and monoclonal antibodies) in different combinations are examples of adjuvant treatment [19]. To enhance patient outcomes, more interventional studies evaluating innovative immunotherapy combinations are required [16]. Tamoxifen and other hormone therapies have occasionally been used. Isolated metastases have also been treated with local radiotherapy [4].

Gynecologic melanomas have a far worse prognosis than cutaneous melanomas. Compared to ovarian carcinomas at similar stages, the prognosis for primary ovarian malignant melanomas is worse [19]. Patients with ascites, adhesions, full-thickness cyst wall invasion, macroscopic dissemination, and cyst rupture generally have a poor outcome [6]. In cases of POM, it has been demonstrated that the Clark/Breslow staging system for dermoid-associated melanoma offers a more accurate prognosis estimate than the Federation of Obstetrics and Gynecology staging [19]. 

## Conclusions

We present a very uncommon case of a post-menopausal woman with pelvic organ prolapse and a 5-cm primary ovarian malignant melanoma. The management challenges of this patient included a lack of radiological characteristics in the TVUS and MRI scans, with non-specific morphology of the lesion and normal serum tumor markers. Moreover, findings in post-operative clinical and radiological assessment in order to exclude an extraovarian primary melanoma were absent. Finally, there was a lack of a set treatment plan, as there are no specific guidelines for POM.

The patient underwent a LAVH with BSO and received adjuvant immunotherapy, being in good clinical condition 12 months after initial diagnosis. As these incidents are isolated and infrequent, an international registry and database of treatment and outcome will enhance our understanding of the biological behavior of POM and help in establishing therapeutic guidelines.
